# Comparative Study on Epstein-Barr Virus-Positive Mucocutaneous Ulcer and Methotrexate-Associated Lymphoproliferative Disorders Developed in the Oral Mucosa: A Case Series of 10 Patients and Literature Review

**DOI:** 10.3390/diagnostics11081375

**Published:** 2021-07-30

**Authors:** Kyoichi Obata, Tatsuo Okui, Sawako Ono, Koki Umemori, Shoji Ryumon, Kisho Ono, Mayumi Yao, Norie Yoshioka, Soichiro Ibaragi, Akira Sasaki

**Affiliations:** 1 Department of Oral and Maxillofacial Surgery, Okayama University Graduate School of Medicine, Dentistry and Pharmaceutical Sciences, 2-5-1, Shikata, Okayama-City 700-8558, Japan; kkume51@gmail.com (K.U.); skutuku@yahoo.co.jp (S.R.); de20012@s.okayama-u.ac.jp (K.O.); noriy@md.okayama-u.ac.jp (N.Y.); sibaragi@md.okayama-u.ac.jp (S.I.); aksasaki@md.okayama-u.ac.jp (A.S.); 2 Department of Oral and Maxillofacial Surgery, Shimane University Faculty of Medicine, 89-1, Enya, Izumo-City 693-8501, Japan; 3 Department of Pathology, Kagawa Prefectural Central Hospital, 1-2-1, Asahi, Takamatsu-City 760-8557, Japan; de19008@s.okayama-u.ac.jp; 4 Department of Dentistry and Dental Surgery, Tsuyama Chuo Hospital, 1756, Kawasaki, Tsuyama-City 708-0841, Japan; myao0120@gmail.com

**Keywords:** methotrexate, lymphoproliferative disorders, Epstein-Barr virus, mucocutaneous ulcer, rheumatoid arthritis

## Abstract

Methotrexate-associated lymphoproliferative disorder (MTX-LPD) is an iatrogenic immunodeficiency-associated lymphoproliferative disorder that occurs mainly with MTX use. This disorder has been associated with Epstein-Barr virus (EBV) infection. In 2017, the WHO newly defined the disease concept of EBV-positive mucocutaneous ulcer (EBV-MCU) as a good-prognosis EBV-related disease. Here, we report 10 cases of MTX-LPD or EBV-MCU in the oral mucosa. This retrospective, observational study was conducted with MTX-LPD or EBV-MCU in the oral mucosa patients who visited us during the nine year period from 2012 to 2021. We gathered the basic information, underlying disease, histopathological evaluation, treatment and prognosis for the subjects. All were being treated with MTX for rheumatoid arthritis. EBV infection was positive in all cases by immunohistochemistry. A complete or partial response was obtained in all cases with the withdrawal of MTX. Our results suggests that the most common risk factor for developing EBV-MCU is the use of immunosuppressive drugs. The most common site of onset is the oral mucosa, which may be attributed to the mode of EBV infection and the high incidence of chronic irritation of the oral mucosa. A small number of patients had been diagnosed with MTX-LPD, but we consider that these cases were EBV-MCU based on our study.

## 1. Introduction

Methotrexate (MTX) is an immunosuppressive antifolate that was approved as a therapeutic agent for rheumatoid arthritis (RA) by the FDA in 1988. MTX is the anchor drug in RA treatment because of its high efficacy rate, continuation rate, and inhibitory effect on bone destruction and improvement of quality of life [1,2]. However, MTX also has various side effects, such as bone marrow suppression, increased risk of interstitial pneumonia, and gastrointestinal tract disorders [1,3,4,5]. In 1991, Ellman et al. reported MTX-associated lymphoproliferative disorder (MTX-LPD) as a lymphoma that occurred in RA patients using MTX, with more than 100 cases reported at that time [6]. In 2008, MTX-LPD was classified as one of the “other iatrogenic immunodeficiency-associated lymphoproliferative disorders” in the WHO Classification of Tumors of Haematopoietic and Lymphoid Tissue [7]. The progression of RA is considered to be a risk factor for the onset of MTX-LPD, however, the pathogenesis of MTX-LPD remains poorly understood [8].

Epstein-Barr virus (EBV) was detected in approximately half of MTX-LPD cases by blood exam, immunohistochemistry, and in situ hybridization. Latently EBV-infected B-cells, which are normally suppressed, are thought to be reactivated by the immunosuppressive action of MTX, allowing MTX-LPD to develop in EBV-infected patients [9]. EBV-infected MTX-LPD patients usually have a good prognosis upon withdrawal of MTX. Dojcinov et al. reported EBV-positive mucocutaneous ulcer (EBV-MCU) as a good-prognosis EBV-related disease that can be histopathologically diagnosed as a lymphoma with ulcers localized to the skin and mucosa [10]. In 2017, EBV-MCU was newly added to the WHO Classification [11]. The use of immunosuppressive drugs is a major risk factor for the development of EBV-MCU and MTX is the most frequently reported causal agent [12]. Immunosenescence due to ageing and primary immunodeficiency are also known risk factors of EBV-MCU. Currently, reports of EBV-MCU and MTX-LPD with lesions in the oral mucosa are increasing. However, there are few studies that have aggregated and compared MTX-LPD and EBV-MCU in the oral mucosa. In addition, it is not clear how the pathogenesis and pathophysiology of MTX-LPD and EBV-MCU differ in the oral mucosa. Also, MTX-LPD and EBV-MCU does not occur in all patients with risk factors and it is reasonable to assume that there are some additional risk factors that have yet to be discovered.

Here, we report our experience with the treatment of EBV-MCU and MTX-LPD in the oral mucosa and provide a comprehensive review of the clinical features.

## 2. Materials and Methods

This case series was composed of 10 patients with MTX-LPD or EBV-MCU occurring in the oral mucosa who visited the Department of Oral and Maxillofacial Surgery, Okayama University Hospital (Okayama, Japan) and the Department of Dentistry and Oral Surgery, Tsuyama Chuo Hospital (Okayama, Japan) during the nine year period from 2012 to 2021. The patients were identified retrospectively and evaluated for age, sex, autoimmune disease and immunosuppressive therapy details, site of onset, EBV infection, histological findings, treatment, and prognosis.

## 3. Results

Table 1 summarizes the detailed characteristics of 10 patients with MTX-LPD and EBV-MCU in our department and at Tsuyama Chuo Hospital. The average age of patients was 75.1 ± 8.05 years (range 58–87 years) and the male-to-female ratio was 1:4, revealing a predominance of females. Eight patients were referred from general dentists (Case No. 1–6, 9, 10), one was referred from the general hospital dentist (Case No. 8), and one was referred from other departments in our hospital (Case No. 7). All patients were receiving immunosuppressive therapy with MTX for RA; one had an additional autoimmune disease of interstitial pneumonia (Case No. 6). The average period since the diagnosis of RA was 20.0 years (range 5–40 years), and the duration of MTX treatment was 12.7 years (range 5–30 years). Immunosuppressive agents used other than MTX were as follows: prednisolone (PSL) in five patients (Case No. 1, 3, 4, 8, 9), tacrolimus (TAC) in two patients (Case No. 5, 6), and bucillamine in one patient (Case No. 7). The primary site was the gingiva in all cases and the maxillary to mandibular ratio was 1:1 (maxillary: Case No. 2–5, 9; mandibular: Case No. 1, 6–8, 10). One patient had a lesion on the lung as well as the oral mucosa (Case No. 3). All patients were confirmed to be infected with EBV by blood test and histopathological examination. In all cases, partial biopsy under local anesthesia was performed to suspect malignancy. Immunohistochemistry staining showed CD20-positive atypical large lymphocytes in all cases; CD3, CD5, and CD10-positive lymphocytes were not detected in many cases. EBV-encoded small RNA in situ hybridization (EBER-ISH) showed positivity in all cases. Therefore, the histopathological diagnosis was diffuse large B-cell lymphoma with EBV infection in all cases. All patients achieved complete clinical remission with discontinued MTX, without chemotherapy or radiotherapy. Tacrolimus was initiated in two patients after withdrawal of MTX to prevent reactivation of RA (Case No. 1, 4). Below, we present two cases as typical examples.

### 3.1. Case 1

**Figure 1:**
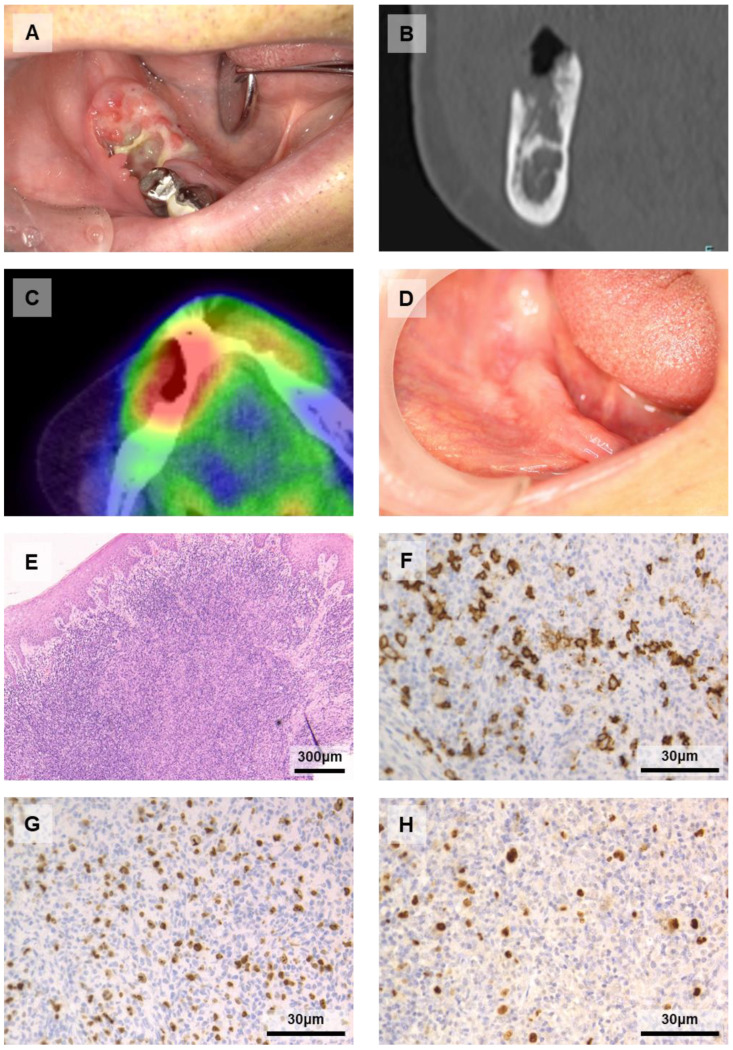
Case 1. (**A**) Pre-treatment intraoral photograph: necrotic bone was exposed in the right mandibular first molar extraction wound with redness and ulceration in the surrounding gingiva. (**B**) Coronal CT: bone destruction of the mandible. (**C**) Horizontal FDG-PET/CT: positive uptake around the lesion. (**D**) Post-treatment intraoral photograph: the wound where the necrotic bone was removed became epithelialized and the lesion completely disappeared. (**E**) Hematoxylin and eosin staining showing diffuse proliferation of atypical lymphocytes in the subepithelium. (**F**–**H**) Immunochemistry staining showing positivity for CD20 (**F**), a high Ki-67 labeling index (**G**), and positivity for EBER-ISH (**H**).

The patient was a 75-year-old Japanese man who visited our department complaining of a painful right mandibular gingiva after first molar extraction. He had had RA for 40 years and had been treated with several immunosuppressive drugs, including MTX (8 mg/week for 20 years), PSL (5 mg/day for 10 years), and golimumab (50 mg/month for 3 years). The intraoral examination showed redness and ulceration of the right mandibular gingiva. There was exposure of necrotic bone in the extraction wound (Figure 1A). Head and neck computed tomography (CT) showed bone destruction from perilesional alveolar bone to the mandibular canal (Figure 1B), but no lymph node abnormality was detected. A blood examination revealed EBV infection [EBV viral capsid antigen (EBV-VCA) IgG = 320, IgM < 10, IgA < 10, EBV nuclear antigen (EBNA) = 20] and a high level of soluble interleukin-2 receptor (sIL-2R = 1224). Fluorodeoxyglucose-positron emission tomography/CT (FDG-PET/CT) indicated the presence of increased uptake in the right mandibular (maximum standardized uptake value: SUVmax = 11.9; Figure 1C) and the right superior internal jugular nodes (SUVmax = 3.78). The biopsy material showed diffuse proliferation of atypical lymphocytes in the subepithelium (Figure 1E). Immunohistochemistry staining showed that the atypical large-sized lymphocytes were positive for CD20 (Figure 1F) and negative for CD3, CD5, and CD10; the Ki-67 labeling index was high (Figure 1G). The result of EBER-ISH was positive (Figure 1H) and the histopathological diagnosis was EBV-MCU (Figure 1E–H).

The patient’s primary physician changed MTX to TAC. The lesion decreased and symptoms disappeared within two weeks. FDG-PET/CT at one year since the withdrawal of MTX showed no increased uptake anywhere. At 20 months after the withdrawal of MTX, the patient was in good condition without recurrence (Figure 1D).

### 3.2. Case 5

**Figure 2:**
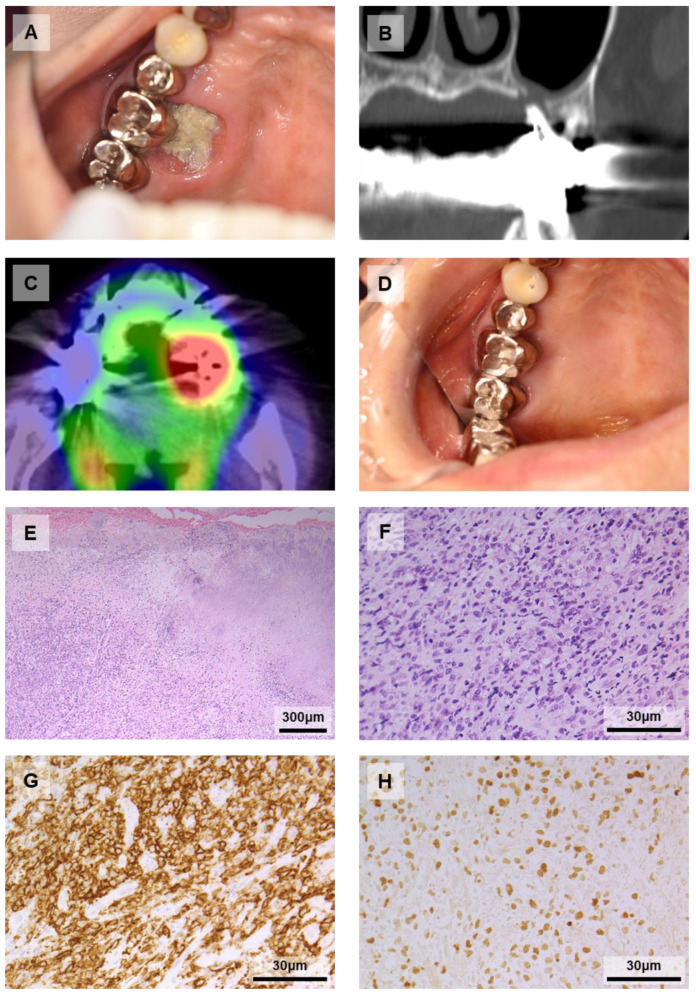
Case 5. (**A**) Pre-treatment intraoral photograph (mirror image): a painless ulceration with bone exposure on the palatal side of the maxillary left first molar. (**B**) Coronal CT: cortical bone destruction on the palatal side. (**C**) Horizontal FDG-PET/CT: positive uptake around the lesion. (**D**) Post-treatment intraoral photograph (mirror image): The ulceration has disappeared and become epithelization. (**E**–**H**) Histopathological findings: Hematoxylin and eosin staining showed diffuse proliferation of atypical lymphocytes with a background of inflammatory cell infiltration (**E**); the lymphocytes were large with prominent nucleoli (**F**). These cells were positive for CD20 (**G**) and EBER-ISH (**H**).

The patient was a 73-year-old Japanese woman who visited our department desiring an examination for bone exposure of the left maxillary gingiva. She had RA for 11 years and had been treated with immunosuppressive drugs, including MTX (14 mg/week for 6 years) and TAC (1.5 mg/day for 1 year). The intraoral examination showed painless ulceration of the gingiva on the palatal side of the first molar with bone exposure (Figure 2A). CT showed cortical bone destruction around the lesion and exposure of the first molar palatal root (Figure 2B). Blood examination for sIL-2R and EBV infection were not performed. FDG-PET/CT indicated only the presence of increased uptake around the lesion (SUVmax = 11.9; Figure 2C). The biopsy material showed diffuse proliferation of lymphocytes with a background of inflammatory cell infiltration (Figure 2E); the lymphocytes were large and atypical (Figure 2F). Immunohistochemistry staining showed that the atypical large-sized lymphocytes were positive for CD20 (Figure 2G), 30, and negative for CD3. EBER-ISH was positive (Figure 2H). The histopathological diagnosis was MTX-LPD (in the current definition, EBV-MCU).

The patient’s primary physician discontinued MTX and the lesion gradually became epithelialized. FDG-PET/CT at seven months after the withdrawal of MTX showed no increased uptake anywhere. At six years after the withdrawal of MTX, the patient was in good condition (Figure 2D).

## 4. Discussion

Although EBV-MCU was newly registered as a subtype of mature B-cell neoplasm in the 2017 WHO classification, it was treated as MTX-LPD until then [7,11]. Lymphoproliferative is a descriptive term that sets this disease group apart from physiological lymphadenopathy and lymphocytosis. The WHO classifies lymphoproliferative disorders (LPD) according to etiology, i.e., LPD associated with primary immune disorders, lymphomas associated with HIV infection, post-transplant LP disorders, and other iatrogenic immunodeficiency-associated LP disorders, including MTX-LPD [7]. Previously, the last of the four classes was identified solely with MTX-LPD because MTX was thought to be the only causative drug for this disease. However, the category has been revised to a broader concept because it was found that immunosuppressive drugs other than MTX can also cause LPD [11,13].

A previous study found that 40–50% of MTX-LPDs develop in extranodal sites, with lesion sites throughout the body, such as the gastrointestinal tract, skin, lung, and head and neck region, including the gingiva, tongue, and mouth floor [7]. EBV infection has been detected in about half of MTX-LPDs and many EBV-positive MTX-LPD cases have shown resolution or reduction of lesions with MTX withdrawal. The prognosis of the latter type of case is relatively good, while cases without response to drug withdrawal require chemotherapy and have poor outcomes [7,11,14].

EBV is a virus belonging to the family of Herpesviridae. More than 90% of infections occur via saliva or sexual secretion in adulthood. Initial infection often occurs in infancy, however, these infections usually only cause mild symptoms, if any. In the case of initial infection after adolescence, infectious mononucleosis with fever, pharyngitis, and generalized lymphadenopathy develops. After initial infection, EBV continues to infect B-cells or epithelial cells of the nasopharynx, although the immune mechanism induces a latent infection state in which virus is not produced. However, in an immunosuppressive state caused by an autoimmune disease or the use of an immunosuppressive drug, reactivation of latent EBV-infected B-cells and subsequent proliferation of viral particles induces various diseases. Diseases caused by reactivation of EBV-infected B-cells include chronic active EBV infection and EBV-positive diffuse large B-cell lymphoma; EBV-MCU is also one of these pathological conditions [15,16].

In terms of research on EBV-MCU, Sinit reported that the most common risk factor for developing EBV-MCU was the use of immunosuppressive drugs, with MTX being the most commonly associated drug (46%), followed by azathioprine, mycophenolate, prednisone, rituximab, and cyclosporine A. The most common reason for immunosuppressive drug use was autoimmune disease (64%), followed by organ or stem-cell transplantation and hematologic malignancies. The most common scenario was an RA patient being treated with MTX (20% of cases). The sites of onset were the oral and oropharyngeal mucosa, gastrointestinal tract, and skin [12]. The high frequency of onset in the oral mucosa was attributed to the mode of EBV infection (lytic vs. latent infection) and the chronic irritation associated with colonization by anaerobic bacteria. During lytic infection, EBV easily colonizes oral mucosa. In this infection mode, the infected B-cells sequestered in the lymphoid tissues of Waldeyer’s ring, etc., are activated due to immunosuppression. After cell death, the lysed B-cells release infectious viral particles, and the mucosal epithelial cells of the oral cavity and ductal epithelial cells of the salivary gland become infected. In addition, the oral mucosa is often chronically irritated by dental caries, periodontal disease, inadequate crown-bridge restorations, dentures, occlusion, etc., leading to the occurrence of cell-level microtrauma. Due to these factors, EBV-MCU is thought to occur frequently in the oral mucosa [12,17,18]. We conjectured that EBV-MCU may be triggered by immunosuppression caused by MTX and that oral peculiarities may be strongly involved in the development of the disease. A disease with a similar pathogenic mechanism is Merkel cell carcinoma. Merkel cell carcinoma has been demonstrated to be triggered by iatrogenic immunosuppression due to the biologics employed in autoimmune diseases therapy, and Merkel cell polyomavirus is reactivated when certain conditions are combined [19]. Therefore, EBV-MCU does not occur in all patients using MTX. We suspect that risk factors such as the degree of infection of the mucosal epithelial cells of the oral cavity and ductal epithelial cells of the salivary gland, and the presence of chronic irritation of the oral mucosa, are strongly involved in the development of EBV-MCU.

When MTX-LPD or EBV-MCU develop, treatment for autoimmune disease becomes difficult because immunosuppressive drugs need to be withdrawn or changed. In particular, after the withdrawal of MTX in RA patients, the majority of patients experience a reactivation of RA. The latest guidelines suggest the use of rituximab or tacrolimus for patients with RA reactivation [20]. Satou et al. reported that rituximab may be effective to prevent regrowth of EBV-MCU [21]. MTX was withdrawn in all 10 cases we experienced, and although RA worsened in some patients, it was controlled with rituximab or tacrolimus.

The 10 cases we experienced had the following characteristics. (1) All patients were being treated for RA with MTX. Immunosuppressive drugs used in combination with MTX included prednisolone. (2) EBV infection was positive in all cases by in situ hybridization and, in some cases, by blood test. (3) In all patients, withdrawal of immunosuppressive drugs without chemotherapy or surgery was selected. (4) Regarding prognosis, a complete or partial response was obtained in all cases without additional treatment. A total of three patients were diagnosed with MTX-LPD before the WHO approved the concept of EBV-MCU. However, we determined that all of the cases could be considered EBV-MCU. This is supported by the report of Yamakawa et al. that 42% of MTX-LPD patients and 13% of elderly LPD patients among all LPD patients are rediagnosed as having EBV-MCU [9]. We also previously reported that cases diagnosed as MTX-LPD in the oral mucosa were highly likely to be EBV-MCU because such cases had a higher rate of EBV infection, a lower rate of ectopic cases, and a better prognosis than MTX-LPD at other sites in the body [22]. Therefore, we considered that most of the LPDs with EBV infection that develop in the oral mucosa of patients using MTX are likely to be EBV-MCU. However, despite speculation that various factors are involved in the onset of LPD and that they interact in a complicated manner, few studies pursuing this possibility have been performed. It is a topic worthy of future research.

## 5. Conclusions

All the patients we encountered were being treated for RA using MTX and were positive for EBV infection. In addition, all patients had a good prognosis with the withdrawal of immunosuppressive drugs. We consider that EBV-positive LPD in the oral mucosa of a patient using MTX is likely to be EBV-MCU.

## Figures and Tables

**Table 1:** Characteristics of patients and course with MTX-LPD or EBV-MCU occurring in the oral mucosa.

No	Age	Sex	Autoimmune Disease			Immunosuppressive Therapy			
			RA	Other	Duration (Year)	MTX	Dose (mg/week)	Duration (Year)	Other
1	75	M	+	-	40	+	8	>20	PSL
2	80	M	+	-	N/A	+	6–8	6	-
3	58	F	+	-	10	+	2–4	10	PSL
4	67	F	+	-	28	+	N/A	25	PSL
5	73	F	+	-	11	+	14	6	TAC
6	74	F	+	IP	12	+	10	11	TAC
7	77	F	+	-	24	+	10	11	BUC
8	79	F	+	-	40	+	2.5	30	PSL
9	81	F	+	-	5	+	8	5	PSL
10	87	F	+	-	10	+	6	10	-
**Primary** **Site**		**Multiple** **Site**		**EBV** **Infection**	**Immunophenotype**		**Histology**	**Therapy** **and** **Response**	**Follow Up** **Period** **(Month)**
					**Positive**	**Negative**			
Mandibular gingiva		-		+	CD20	CD3, 5	DLBCL	WM -> CR	24
Maxillary gingiva		-		+	CD20	N/A	DLBCL	WM -> CR	92
Maxillary gingiva		+ (lung)		+	CD20	CD3, 5, 10	DLBCL	WM -> CR	36
Maxillary gingiva		-		+	CD20	CD3, 5, 10	DLBCL	WM -> CR	30
Maxillary gingiva		-		+	CD20, 30	CD3	DLBCL	WM -> CR	27
Mandibular gingiva		-		+	CD20	CD3, 5, 10	DLBCL	WM -> CR	20
Mandibular gingiva		-		+	CD20, 79a	CD3, 5	DLBCL	WM -> CR	13
Mandibular gingiva		-		+	CD20, 79a	CD3, 5	DLBCL	WM -> CR	7
Maxillary gingiva		-		+	CD20, 79a	CD3, 5	DLBCL	WM -> CR	31
Mandibular gingiva		-		+	CD20	CD3, 5, 10	DLBCL	WM -> CR	12

BUC = bucillamine, CR = complete remission, DLBCL = diffuse large B-cell lymphoma, EBV = Epstein-Barr virus, IP = interstitial pneumonia, MTX = methotrexate, PSL = prednisolone, RA = rheumatoid arthritis, TAC = tacrolimus, WM = withdrawal of MTX.

## Data Availability

All supporting data are provided in the current manuscript.

## Abbreviations

CT	computed tomography
EBER-ISH	Epstein-Barr virus-encoded small RNA in situ hybridization
EBNA	Epstein-Barr virus nuclear antigen
EBV	Epstein-Barr virus
EBV-MCU	Epstein-Barr virus-positive mucocutaneous ulcer
EBV-VCA	Epstein-Barr virus capsid antigen
FDG-PET/CT	fluorodeoxyglucose-positron emission tomography/computed tomography
LPD	lymphoproliferative disorders
MTX	methotrexate
MTX-LPD	methotrexate-associated lymphoproliferative disorders
PSL	prednisolone
RA	rheumatoid arthritis
sIL-2R	soluble interleukin-2 receptor
SUVmax	maximum standardized uptake value
TAC	tacrolimus
